# Response to spectral-domain optical coherence tomography foveal morphology as a prognostic factor for vision performance in congenital aniridia

**DOI:** 10.1177/1120672120912658

**Published:** 2020-03-16

**Authors:** Sohaib R Rufai, Mervyn G Thomas, Irene Gottlob

**Affiliations:** 1 The University of Leicester Ulverscroft Eye Unit, Leicester Royal Infirmary, Leicester, UK; 2 Clinical and Academic Department of Ophthalmology, Great Ormond Street Hospital for Children NHS Foundation Trust, London, UK

Dear Editor

We read with interest the study published by Casas-Llera et al.^1^ The authors have correlated best-corrected visual acuity (BCVA) with the structural grading system for foveal hypoplasia developed by our unit.^2^ Recently, as recommended by Wilk et al.,^3^ we have updated the grading system to include two subsets of grade 1 foveal hypoplasia: grade 1a, in which nearly normal pit metrics are observed, and grade 1b, in which the pit is only a shallow indent. We recently published our updated and validated grading system, demonstrating that it can predict future vision in infants and young children with foveal hypoplasia.^4^ The subsets of grade 1 do not apply to the cohort reported by Casas-Llera et al., as they only detected grade 2–4 foveal hypoplasia, furthermore we note that their paper was submitted before our latest paper was published. Interestingly, a previous paper by Sannan et al.^5^ reported a full spectrum of grade 0–4 foveal hypoplasia in a large cohort of PAX6 mutations.

Casas-Llera et al. state that the minimum age they considered appropriate to collaborate with optical coherence tomography (OCT) was 4 years. We would like to highlight that handheld OCT is possible in awake infants with nystagmus from birth in our experience, without sedation or dilation, such as in our latest study with infants as young as 28 days old.^4^ We accept that a great number of eye departments do not have access to or experience in handheld OCT imaging, therefore it was reasonable for the authors to exclude children too young to cooperate with conventional OCT.

Their study demonstrated that higher foveal hypoplasia grades correlated with poorer BCVA, consistent with findings from our unit and others.^2,4^ It would be highly valuable to assess whether BCVA in the younger children improved further over time. Our recent prediction study included OCT data for preverbal infants and young children at first examination, then BCVA was recorded when the child was old enough to participate in gold standard LogMAR chart testing.^4^ Moreover, in the study by Casas-Llera et al., we wonder whether cataracts could have influenced BCVA? Or perhaps the subcapsular cataracts were not in the visual axis?

Casas-Llera et al. comment that their population excludes children aged below 4 years, avoiding misclassifications due to physiological development. However, despite continued foveal development in early childhood, we have demonstrated that all grades of foveal hypoplasia remained stable in our cohort,^4^ owing to the qualitative nature of the grading system. We believe that misclassification is only possible between grades 2 and 3 in the first 6 months of life, when outer segment elongation takes place.

Finally, we strongly agree with Casas-Llera et al. in their comment regarding the clinical value of foveal hypoplasia grading to inform investigation and management. If the grade of foveal hypoplasia is mild but the BCVA disproportionately poor, suspicion should be raised for other factors limiting BCVA which require appropriate investigation and management.
